# The Puzzle of the Walnut Shell: A Novel Cell Type with Interlocked Packing

**DOI:** 10.1002/advs.201900644

**Published:** 2019-06-11

**Authors:** Sebastian J. Antreich, Nannan Xiao, Jessica C. Huss, Nils Horbelt, Michaela Eder, Richard Weinkamer, Notburga Gierlinger

**Affiliations:** ^1^ Department of Nanobiotechnology University of Natural Resources and Life Sciences Vienna (BOKU) 1190 Vienna Austria; ^2^ Department of Biomaterials Max‐Planck Institute of Colloids and Interfaces Science Park Potsdam‐Golm 14424 Potsdam Germany

**Keywords:** 3D structures, interlocked packing, puzzle cells, sclereids, walnut shell

## Abstract

The outer protective shells of nuts can have remarkable toughness and strength, which are typically achieved by a layered arrangement of sclerenchyma cells and fibers with a polygonal form. Here, the tissue structure of walnut shells is analyzed in depth, revealing that the shells consist of a single, never reported cell type: the polylobate sclereid cells. These irregularly lobed cells with concave and convex parts are on average interlocked with 14 neighboring cells. The result is an intricate arrangement that cannot be disassembled when conceived as a 3D puzzle. Mechanical testing reveals a significantly higher ultimate tensile strength of the interlocked walnut cell tissue compared to the sclerenchyma tissue of a pine seed coat lacking the lobed cell structure. The higher strength value of the walnut shell is explained by the observation that the crack cannot simply detach intact cells but has to cut through the lobes due to the interlocking. Understanding the identified nutshell structure and its development will inspire biomimetic material design and packaging concepts. Furthermore, these unique unit cells might be of special interest for utilizing nutshells in terms of food waste valorization, considering that walnuts are the most widespread tree nuts in the world.

One of the most important evolutionary success stories in plants is the development of seeds encased in maternal tissue, such as fruit walls and seed coats.^1^ These outer protective layers can show remarkable toughness and strength, which has attracted considerable attention related to their microstructure, particularly with the aim to develop biomimetic materials.^2^, ^3^, ^4^ In this study, we reveal the 3D micro‐ and nanostructure of the walnut shell over the course of its development, from the soft to the hard state. Besides light and electron microscopic investigations, 3D visualizations of the tissue and chemically isolated single cells were the key to identify the novel cell morphology and its interlocked packing.

On the microscale, the walnut (*Juglans regia*) shell is characterized by a dense tissue, in which single cells and their shape are difficult to identify – even at higher magnification (**Figure** **1**a). Only in the inner part of the shell (bottom of the image) the tissue structure is looser and thin walled cells are visible: small roundish ones together with very big ones and irregular lobes (Figure 1a). Prior to maturity (harvested in July, June, and May), the developing nutshell is surrounded by a green husk (Figure 1b) and possesses a loose tissue structure throughout the entire shell. In July, the polylobate tissue structure is clearly visible and with a circularity of 0.43 similar to that in October. In June, smaller cells with less lobes (circularity = 0.49) dominate and the cells in the youngest and smallest nuts (sampled in May) had not yet developed lobes (Figure 1b). The polygonal cell shape in the beginning of the development of the walnut shell resembles more the cell shape in a mature pine (*Pinus koraiensis*) seed coat (Figure 1c). However, cells have thick walls as typical for sclerenchyma cells and are found in the entire seed coat as the only cell type (Figure 1c). Other nutshells, like Macadamia, show additionally to the isodiametric sclerenchyma cells also fibrous cells.^2^, ^3^, ^4^, ^5^ What all sclereid cells have in common is a multilayered cell wall structure,^4^, ^5^ which apparently results from a helicoidal arrangement of the cellulose microfibrils.^6^

**Figure 1:**
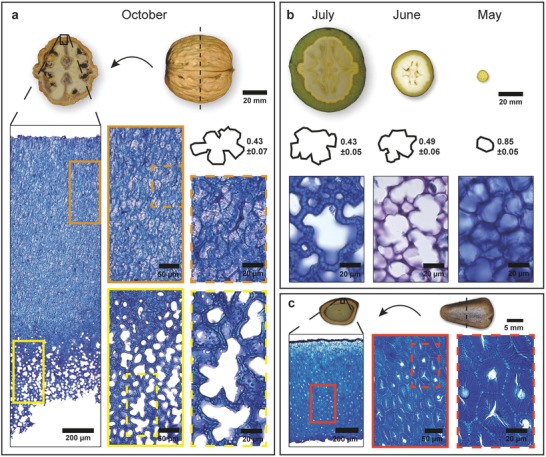
Tissue structure of walnut shell and pine seed coat. a) The mature walnut, harvested in October, shows a brown and fully developed shell. Toluidin blue stained microsection reveals a dense outer layer and an inner layer with thin‐walled cells, exhibiting the polylobate cell shape. b) The developmental stages of a walnut with strong increase of the fruit from May to July. The polylobate shape in June and July with high circularity derives from a regular cell shape and cell arrangement in May. c) In comparison, the dense tissue structure from a mature pine seed coat with nonlobate cells.

In order to investigate the cell shape in more detail, single cells were isolated from the tissue composite by lignin removal (maceration) and imaged via confocal laser scanning microscopy (CLSM; **Figure** **2**a, Movie S1, Supporting Information) and electron microscopy (Figure S1, Supporting Information). Two polylobate cells are visualized in different depths: several slices of the *z*‐stack clearly show that they are indeed single cells with lobes (Figure 2a, 3–4); while, other slices through the lobes might give a false impression of being single cells (Figure 2a 1–2,5–6). Based on the *z*‐stack, a model of the two polylobate cells was reconstructed, which shows, in addition to the lobes, that many pores perforate the cell wall (Figure 2a). These pores play an essential role for exchanging chemical compounds during growth and development. The thinner the cell wall (early stage, inner part), the bigger the pores are (Figure 2a, right upper cell compared to thicker walled cell in the left corner).

**Figure 2:**
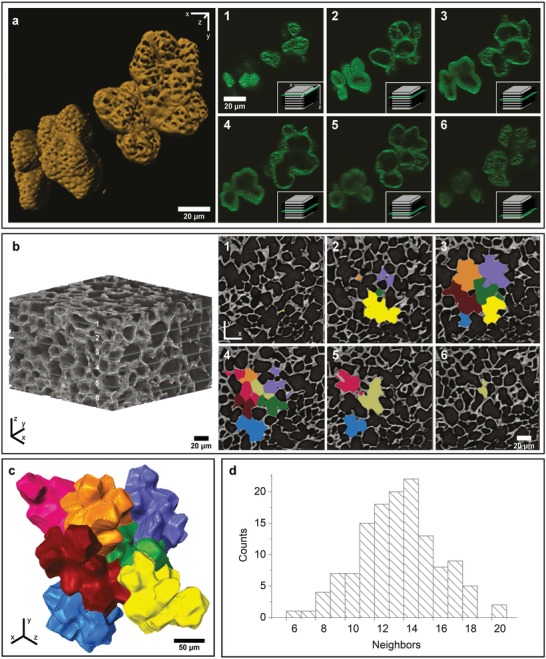
3D visualization of walnut cells by CLSM and nano‐CT. a) Six exemplary slices of the CLSM‐scan (100 slices, see also Movie S1 in the Supporting Information) of two single cells, stained with Calcofluor White. The two cells show a difference in the cell wall thickness (best seen in 2 and 3). Icons on the bottom right show the position of the slice in the stack. 3D reconstruction of the same CLSM‐scan, revealing the lobed shape of the single cells and the high number of pores, which are bigger in the thin walled (upper right cell) compared to the thicker walled cell (bottom left). b) Orientation and arrangement of the shell piece, which consists of 170 slices in *z*‐direction; six slices are marked in green and shown on the right (1–6). The colored areas indicate the individual cells used for digital segmentation and reconstruction. c) 3D reconstruction of the segmented cells, showing the interlocking between neighboring cells (see also Movie S2 in the Supporting Information). d) The distribution of neighboring cells after analyzing the contact areas between the singles cells after 3D reconstruction.

The 3D arrangement of these polylobate cells is confirmed by X‐ray nanotomography of the shell. The segmented cells (Figure 2b) resemble the 2D puzzle‐shaped epidermal cells of *Arabidopsis thaliana* leaves and cotyledons.^7^, ^8^, ^9^ However, when the segmented cells are reconstructed, the jigsaw puzzle is revealed for the first time in 3D (Figure 2c). Each of the polylobate cells is interlocked on average with 14 neighboring cells (Figure 2d and Movie S2, Supporting Information).

Based on the number of nearest neighbors (Figure 2d) and the cells observed at early developmental stages (Figure 1b), we suggest that the cells initially started form an arrangement resembling a tetrakaidecahedron configuration, also used for describing and modeling cellular solids.^10^ The processes that underlie the formation of puzzle cells in *Arabidopsis* leaves and cotyledons have been a hot research topic over the last 2 decades eg.^7^, ^8^, ^9^ The functional benefit of the complex shell shape has remained elusive. However, it was recently proposed that these intricate forms provide an effective strategy to reduce mechanical stress in the cell wall of the epidermis.^8^ It was shown that cell shape influences the direction and magnitude of mechanical stress exerted on the cell wall, and that the driving force of the complex puzzle shape originates from growth restriction in the indentations rather than promoting growth in the protrusions.^8^ To understand the development of the complex 3D puzzle shaped structure in walnut shells from first principles, studies on the microtubule trajectories and cellulose orientation in the early developmental stages will be necessary, as well as modeling of growth. If mechanical stress and constraint are the two main driving factors, biomimetic fabrication routes might be derived by using swellable material components.

To quantify how the presence of lobes affects the mechanical performance, pine seed coats were chosen for comparison. This selection is based on a “sclerenchyma screening,” which showed that the pine seed coat is also composed of thick‐walled sclerenchyma cells only, however, without lobes (Figure 1c). For a mathematical description of the differences the volume to surface area ratio was calculated based on X‐ray tomography data for walnut cells from July and October samples and compared to pine cells (**Figure** **3**a). The polylobate cell form of the walnut shell implies a 30–40% higher surface area compared to the polygonal cell form of the pine seed coat. The latter come close to the trend line of the tetrakaidecahedron (Figure 3a), which can be seen as the basic form for tissue packaging with 14 faces. Micromechanical testing of the walnut shell resulted in more scattered data compared to the pine seed coat (Figure 3b). This is explained by the fact that different proportions of the less dense inner tissue were present in the tested walnut sample, while the pine seed coat was uniform throughout in terms of density (Figure 1, Figure 3c,d). Mechanical testing of walnut shells yielded a median Young's modulus of 5.2 GPa and a median ultimate tensile strength of 51.1 MPa. Pine seed coats show a comparable stiffness (median: 4.4 GPa); however, they demonstrated a significantly lower ultimate tensile strength (38.5 MPa) (Figure 3b). The fracture surface of the pine seed coat reveals more intact cell surfaces due to rupture of the interface between the cells (Figure 3c). In contrast, the walnut shells often show fractures across the lobes due to the interlocking (Figure 3d). Similarly, in the inner part of the walnut shell with thin‐walled cells, more fractures are observed cutting through the whole cell, thereby opening the view on the big polylobate cell form. (Figure 3d, right image). The higher surface area and interlocking explain the higher ultimate tensile strength, while the Young's modulus is similar due to the same “material” (cell wall structure and composition).

**Figure 3:**
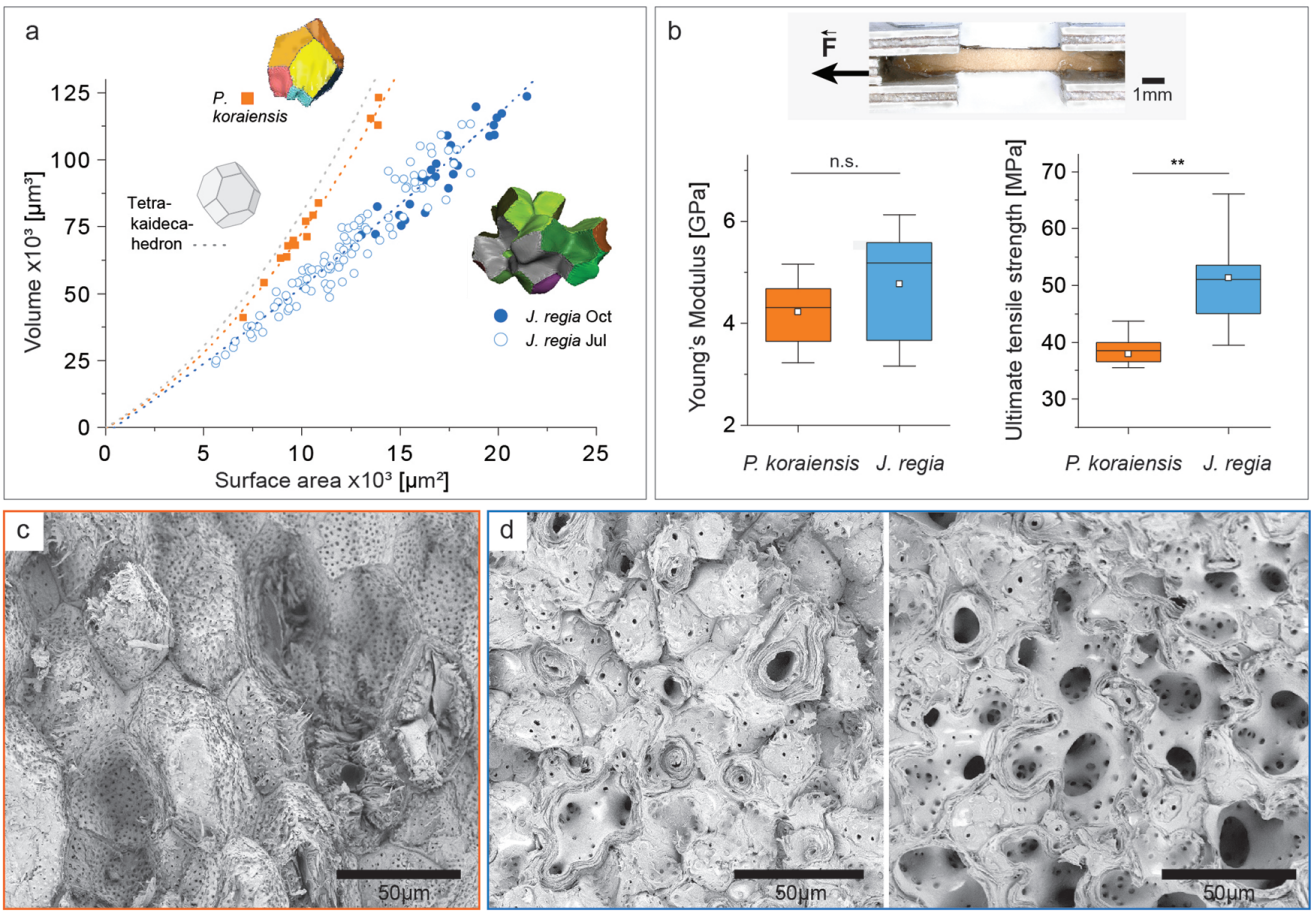
Comparison of cell shape and mechanics of walnut shells and pine seed coats. a) Volume to surface area ratio for single walnut cells from July and October samples, compared to pine cells and the tetrakaidecahedron, which is the basic form for tissue packaging with 14 faces. b) Exemplary tensile test sample and mechanical data (Young's modulus and ultimate tensile strength) for walnut shell (sampled in October, *n* = 10) and pine seed coat (*n* = 8). c) SEM image of the fracture surface after tensile testing of pine and d) walnut (example for dense outer part on the left and inner part on the right side).

The interdigitated arrangement of walnut cells (Figure 2c, Movie S2, Supporting Information) is a fascinating example of smart geometrical arrangements in biological materials, which can be classified as a tessellation. This structural motif involves periodic arrangement of soft and hard elements in series, which has been reported at all scales; from molecular arrangements to macroscopic units for a vast array of biological materials.^11^, ^12^ For plants, a wavy sutural tessellation was recently reported in the seed coat of the common millet (*Panicum miliaceum*) as a strategy to amplify strength, toughness, and auxeticity.^13^ In material design, the idea of tessellation is realized in interlocking materials to achieve high resistance to crack propagation, large energy absorption capacity, and remarkable tolerance to local failures.^14^, ^15^ These materials are based on building blocks of exactly the same geometry and rely on an outer border as a constraint. Our reported 3D puzzle structure is interlocked individually; many different structured lobes are fitted into their negative neighbors and will function without any border constraint.

Walnut shells are an abundant by‐product of the food industry,^16^ which are usually burned although more and more potential valorization applications are emerging.^17^ Considering the promising approaches for new high‐performance structural and functional materials based on natural wood,^18^, ^19^, ^20^, ^21^ walnut shells also might be interesting for densification, as well as impregnation and molding into structures with other biopolymers for a more sustainable bioeconomy.

## Experimental Section

*Sampling*: Walnuts (*Juglans regia*, cultivar “Geisenheim”) were collected from July to October 2017 and from Mai to October 2018 from a 48‐year‐old tree in the “BOKU horticulture Jedlersdorf” in Vienna, Austria. For each sampling, ten nuts were randomly collected and immediately stored in plastic bags at −20 °C until further investigation. The nuts and their cross‐sections were photographed with a Canon EOS M10 with a macro lens (35 mm, f/2.8). Pine nuts (*Pinus koraiensis*) were grown in northeastern China near the Changbai mountains and harvested in 2018.

*Histological Staining*: Toluidine Blue O (Sigma‐Aldrich) and Euparal (ROTH) were used to stain 8 µm thin sections, originating from blocks of the frozen walnut sampled in Mai, June and July, and cut with a Cryostat Leica CM 3050 S (Leica) at −10 °C. The October walnut and pine nut samples were cut with a rotary microtome Leica RM 2255 (Leica) after soaking in distilled water for 48 h at room temperature. Sections were stained immediately with Toluidine Blue O solution (*c* = 0.2 mg mL^−1^) and incubated at room temperature for 30 min, and then washed with distilled water until the wash solution is clear. Stained sections were embedded in Euparal and photographed with a Labophot‐2 microscope (NIKON).

*Circularity*: From the different walnut sections, perimeter and area of the biggest cells (*n* = 30) were measured digitally (ImageJ, NIH) and the circularity was calculated with the formula 4 π (area/perimeter2). The higher the ratio, the more the shape approaches a perfect circle characterized by a circularity equal to 1.

*Delignification (Maceration)*: Blocks of walnut shells (avg. 5 mm × 5 mm × 2 mm) harvested in July and October were macerated by immersion in a solution containing H_2_O_2_ (30%, ROTH), distilled water, and acetic acid (>99.8%, Sigma‐Aldrich; V:V:V = 1:4:5), kept in an 1.5 mL Eppendorf tube 72 h in the oven (60 °C), rinsed three times with distilled water, followed by vigorous shaking to separate the single cells.

*Confocal Laser Scanning Microscopy (CLSM)*: For visualizing the macerated single cells, two drops of Calcoflour White (1 g L^−1^, Sigma‐Aldrich) were put on a glass slide, on top of the macerated single cells. A LEICA SP5 was equipped with a 60 × /0.9 water objective, and a diode laser (λ_exc_ = 405 nm). The stained cellulose was detected at 425–475 nm. Images were obtained with a step size of 0.4 µm, covering the entire volume of cells (50–100 planes) via the Leica software (LAS AF 3.1). The software Imaris (8.4.0, Bitplane) was used for 3D reconstruction of the cells.

*Scanning Electron Microscopy (SEM)*: For imaging of the shell tissue and single cells, oven‐dried samples (24 h at 60 °C) were mounted on aluminum stubs with double‐sided sticky carbon tapes and gold sputtered with a sputter coater (LEICA EM SCD005) prior to visualization under a scanning electron microscope (FEI Apreo). Scanning parameters were set to 1.0 kV beam voltage and 6.3 pA current. A work distance between 3.3 and 7.8 mm was chosen depending on the magnification.

*Microcomputed Tomography (CT) and 3D Visualization*: For imaging, nutshells (October, avg. 1 mm × 1 mm × 3 mm) were scanned in an X‐ray microcomputed tomography (RXsolutions EasyTom 160). Scanning parameters of the nanofocus tube (diamond target and tungsten/LaB6 filament) were set to 60 kV tube voltage and 200 µA current (exp. time 0.5 s and frame averaging 3). The exposure time of the flat panel detector was 0.5 s, frame averaging was 3, and the voxel size was 0.8 µm. Each scan consisted of 1268 radiographs, which were reconstructed in the software XAct 2 (RXsolutions) and then visualized in the software Amira (FEI, Version 6.1). Stacks in *z*‐direction were first filtered with nonlocal means, followed by a median filter, and then the cell lumen and the cell wall were selected with the segmentation toolbox. The cell wall was added to each cell and a surface plot was generated to render the volumes in 3D. The cell surface area and the volume, as well as the number of contact areas of neighboring cells, were then calculated from the surface plot of single cells.

*Tensile Testing*: Mature walnut shells collected in October 2018 and pine nuts (*Pinus koraiensis*) were precut with a hand‐saw and trimmed to rectangular bar specimens (width ≈ 1–2 mm, only walnuts shells were trimmed to a uniform thickness of ≈0.90–1.15 mm due to their waviness) with a Cryostat (CM 3050 S, Leica) at −10 °C. For each species, eight to ten test specimens were selected and both ends glued (Loctite 454, Henkel) onto support strips (three‐layered model aircraft plywood from birch, 1 mm thick, cut to a length of 60 mm and a width of 18 mm) with a distance of 4 mm. Two additional support strips were glued (Ponal Express Holzleim, Henkel) on top of the first strip (sandwich structure shown in Figure 3b) to provide additional support. Immediately after the application of the glues, the sandwich construction was compressed for 15 min by placing a weight of ≈2 kg on top. The mechanical tests started 24 h later to ensure glue hardening. Tensile loading was performed with a 2.5 kN testing machine (zwickiLine Z2.5, Zwick‐Roell), equipped with a load cell of 1 kN and a video extensometer (videoXtens, Zwick‐Roell) to measure length changes of the specimen during testing (preload 1 N, test speed 0.004 mms^−1^). The initial clamp distance was set to 45 mm. The dimensions of each specimen were determined with an electronic caliper to calculate the cross‐sectional area. For the calculation of the Young's modulus, the range of 0.02–0.12% strain was used. Statistical analyses were performed in OriginPro by applying the Mann–Whitney test (significance levels: 0.05*; 0.01**).

## Conflict of Interest

The authors declare no conflict of interest.

## Supporting information

Supplementary

Supplementary

Supplementary
