# Supplementary material for: The Puzzle of the Walnut Shell: A Novel Cell Type with Interlocked Packing
Source: Adv Sci (Weinh). 2019 Jun 11;6(16):1900644. doi: 10.1002/advs.201900644 (PMC6702760; doi:10.1002/advs.201900644)
Supplement: Supplementary file 1 — Supplementary [file ADVS-6-1900644-s002.pdf]

Copyright WILEY-VCH Verlag GmbH & Co. KGaA, 69469 Weinheim, Germany, 2019.

ADVANCED SCIENCE
Open Access

Supporting Information

for *Adv. Sci.,* DOI: 10.1002/advs.201900644

The Puzzle of the Walnut Shell: A Novel Cell Type with Interlocked Packing

*Sebastian J. Antreich, Nannan Xiao, Jessica C. Huss, Nils Horbelt, Michaela Eder, Richard Weinkamer, and Notburga Gierlinger**

Copyright WILEY-VCH Verlag GmbH & Co. KGaA, 69469 Weinheim, Germany, 2019.

Supporting Information

The puzzle of the walnut shell: a novel cell type with interlocked packing

*Sebastian J. Antreich, Nannan Xiao, Jessica C. Huss, Nils Horbelt, Michaela Eder, Richard Weinkamer, and Notburga Gierlinger**

Correspondence to: burgi.gierlinger@boku.ac.at

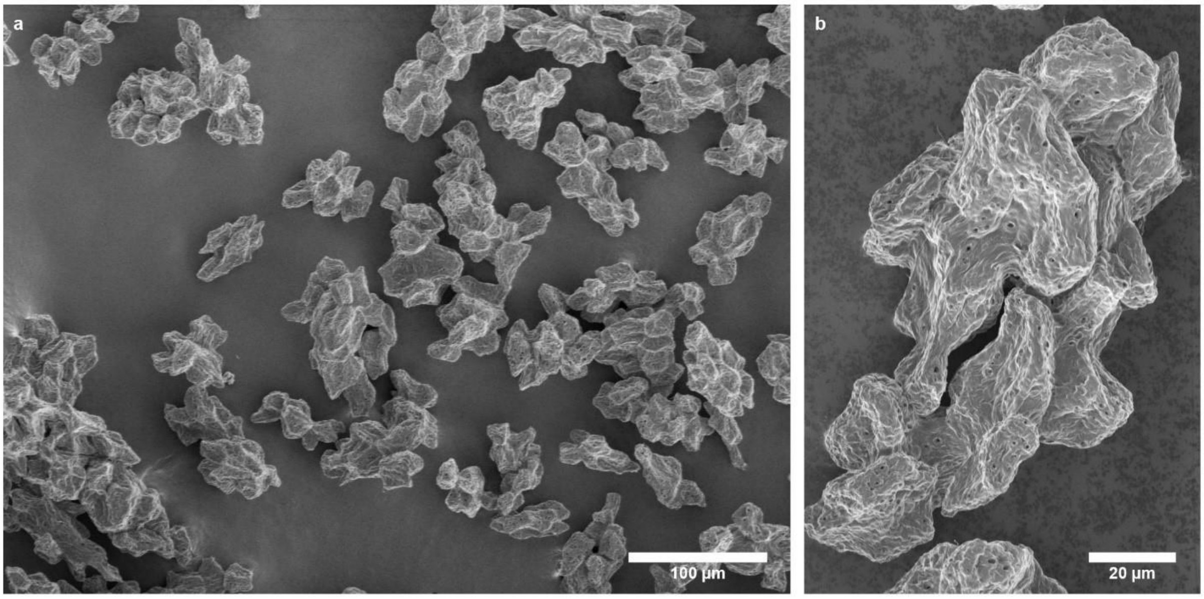

Figure S1. Scanning electron microscopy (SEM) of macerated walnut cells. (a) Diversity of polylobed single cells of a walnut shell (October) after treating with H2O2 and acetic acid (b) a close-up of two interlocked cells.

Movie S1. (*online*) Confocal laser scanning microscope (CLSM) depth scan through two single cells, chemically isolated from the walnut shell and treated with Calcofluor white to stain cellulose.

Movie S2. (*online*) Arrangement of several cells in the walnut tissue after CT-reconstruction. The yellow cell is the initial cell and all its 14 neighboring cells appear successively, which illustrates the 3D-jigsaw morphology and the interlocking of the cells.
